# 基于超高效液相色谱-飞行时间质谱法测定*LAMTOR1*在肝脏炎症恶性转化中调控的代谢物

**DOI:** 10.3724/SP.J.1123.2021.06006

**Published:** 2021-10-08

**Authors:** Wen WANG, Di CHEN, Hailong PIAO

**Affiliations:** 1.中国科学院大连化学物理研究所, 中国科学院分离分析重点实验室, 辽宁 大连 116023; 1. CAS Key Laboratory of Separation Sciences for Analytical Chemistry, Dalian Institute of Chemical Physics, Chinese Academy of Sciences, Dalian 116023, China; 2.中国科学院大学, 北京 100049; 2. University of Chinese Academy of Sciences, Beijing 100049, China

**Keywords:** 液相色谱-质谱, 代谢组学, *LAMTOR1*, 非酒精性脂肪肝炎, 恶性转化, liquid chromatography-mass spectrometry (LC-MS), metabolomics, *LAMTOR1*, non-alcoholic steatohepatitis, malignant transformation

## Abstract

LAMTOR1(晚期胞内体/溶酶体接头蛋白,MAPK以及mTOR激活蛋白1)是机体应对营养压力时重要的调控蛋白之一。公共开放基因表达数据库分析显示*LAMTOR1*在非酒精性脂肪肝炎(NASH)和肝癌中均异常高表达,显示*LAMTOR1*在NASH和肝癌发生发展中发挥重要作用,探索*LAMTOR1*在肝脏炎症恶性转化过程中调控的代谢机制具有重要意义。该研究中小鼠给予蛋氨酸胆碱缺乏(MCD)饮食饲养,肝脏组织的苏木精伊红(HE)染色结果显示小鼠肝脏炎症性损伤的成功构建。接下来利用蛋白免疫印迹实验验证了肝脏组织中*LAMTOR1*基因的特异性敲除以及一些*LAMTOR1*调控的蛋白变化。紧接着开展了基于超高效液相色谱-飞行时间质谱联用的肝脏组织代谢组学分析,以鉴定*LAMTOR1*肝脏特异性调控的重要代谢物。对检测到的134个代谢物进行多变量分析,主成分分析模型显示特异性敲除*LAMTOR1*对小鼠肝脏的代谢过程有明显的扰动。其中45个代谢物发生了显著性变化,表明敲除*LAMTOR1*可引起肝脏多条代谢通路紊乱。进一步分析显示,UDP-乙酰葡萄糖胺(UDP-GlcNAc)、*S*-腺苷蛋氨酸、*S*-腺苷高丝氨酸和三甲基赖氨酸等代谢物在*LAMTOR1*敲除(*LAMTOR1*^LKO^)小鼠中明显上调,说明LAMTOR1与己糖胺合成通路和生物分子甲基化过程可能存在调控关系。另外,9-氧代十八碳二烯酸、二十碳五烯酸(EPA)和二十二碳六烯酸(DHA)等不饱和脂肪酸等代谢物水平在*LAMTOR1*^LKO^小鼠中明显下降。接下来基于公共开放转录组数据库开展了基因表达相关性的预测分析,得到的相关系数*R*表征基因间的调控关系,*R*的绝对值接近或高于0.5属于中强相关,结果提示*LAMTOR1*可能负调控*MAT1A* (*R*=-0.47)基因,同时预测得到*LAMTOR1*与*MGAT*1 (*R*=0.47)和*ADSL* (*R*=0.59)等基因存在正调控关系。该研究将代谢组学方法应用于疾病机制研究,通过鉴定小鼠NASH模型中*LAMTOR1*特异性调控的代谢物,并结合基因表达相关性分析,揭示出*LAMTOR1*基因在非酒精性脂肪肝炎及恶性转化过程中可能调控的重要代谢通路,为后续NASH及NASH转化的肝癌发病机制和治疗研究提供理论基础。

## Abstract (en)

The late endosomal/lysosomal adaptor MAPK and mTOR activator 1 (LAMTOR1) is an important regulator protein in the response to energy stress. Public gene expression data shows that the expression of *LAMTOR1* is abnormally high in nonalcoholic steatohepatitis (NASH) and hepatocellular carcinoma (HCC); hence, *LAMTOR1* may play an important role in the development of NASH and HCC. Therefore, exploring the LAMTOR1 regulatory mechanism in the progression of NASH and malignant transformation of liver inflammation may be crucial for translational medicine. First, a NASH mouse model was established by feeding a methionine choline-deficient (MCD) diet. Hematoxylin-eosin staining of liver tissues showed successful modeling of inflammatory injury in the mouse liver. Immunoblot analysis confirmed LAMTOR1- and *LAMTOR1*-mediated protein expression in *LAMTOR1* specifically depleted mouse livers. Subsequently, metabolic profiling of liver tissues was performed using an ultra-performance liquid chromatography-time-of-flight mass spectrometry strategy. Based on the retention time, *m/z* value, and tandem mass spectra, 134 metabolites were identified. Among these, the levels of 45 metabolite were significantly influenced by hepatic *LAMTOR1* depletion. According to the metabolomics results, uridine diphosphate-*N*-acetylglucosamine (UDP-GlcNAc) was significantly upregulated in *LAMTOR1*-depleted (*LAMTOR1*^LKO^) hepatocyte tissues. As the final product of the hexosamine biosynthetic pathway (HBP), alteration in UDP-GlcNAc levels may regulate *LAMTOR1* and metabolic regulatory genes downstream of HBP. Moreover, there was an obvious increase in the levels of several methylation-related metabolites. Thus, upregulated *S*-adenosylmethionine, *S*-adenosylhomocysteine, and *N*6,*N*6,*N*6-trimethyl-L-lysine indicated that *LAMTOR1* may regulate the process of DNA or protein methylation. In addition, downregulation of 9-oxo-octadecadienoate, eicosapentaenoic acid (EPA), and docosahexaenoic acid (DHA) was also observed in *LAMTOR1*^LKO^ mice liver tissues. Alterations in polyunsaturated fatty acids, such as EPA and DHA, link *LAMTOR1* to inflammatory and immune processes, which are known to play important roles in NASH pathogenesis. These metabolic disorders demonstrated that *LAMTOR1* significantly contributed to the metabolic mechanism of NASH. Furthermore, gene expression correlations were analyzed to interpret the regulatory role of *LAMTOR1* from the perspective of genetic networks. Owing to a paucity of liver whole-transcriptome studies in NASH, correlation analysis was performed based on HCC transcriptome data from public databases. First, a negatively regulated relationship was observed between *LAMTOR1* and *MAT1A* (*R*=-0.47). *MAT1A* encodes methionine adenosyltransferase 1A, an essential enzyme that catalyzes the formation of S-adenosylmethionine. Based on the upregulation of UDP-GlcNAc under hepatocyte *LAMTOR1* depletion, it was predicted that *LAMTOR1* positively influenced *MGAT1* (*R*=0.47), a gene encoding alpha-1,3-mannosyl-glycoprotein 2-beta-*N*-acetylglucosaminyltransferase. Together with changes in succinyladenosine caused by hepatocyte *LAMTOR1* deletion, predicted correlation results showed that *LAMTOR1* may also participate in the pathogenesis through the positive regulatory relationship with *ADSL* (*R*=0.59). The *ADSL* gene provides instructions for making an enzyme called adenylosuccinate lyase, which can dephosphorylate the substrate succinyladenosine. In this study, *LAMTOR1* was identified to specifically regulate multiple key metabolic pathways based on both NASH mouse models and gene expression correlations. These results illustrate the important role of *LAMTOR1* in the progression of NASH and malignant transformation of liver inflammation, which provides a theoretical basis for the diagnosis and treatment of NASH or possible NASH-driven HCC.

非酒精性脂肪肝炎(nonalcoholic steatohepatitis, NASH)因为其纤维化和肝硬化带来的严重肝损伤,被认为是非酒精性脂肪肝病(nonalcoholic fatty liver diseases, NAFLD)进展中的关键阶段^[1]^且可能发展为肝癌(hepatocellular carcinoma, HCC)^[2]^,已成为肝脏相关疾病死亡的重要原因之一。NASH的发生发展与导致肝脏中脂质沉积的肥胖及其代谢综合征有较强的关系^[3]^,而伴随着肥胖以及相关代谢疾病越来越全球化的趋势,NAFLD也已经成为很多国家的重要健康问题^[4]^。研究发现,肝脏特异敲除*Pten*基因的小鼠后续会发生NASH^[5]^,严重者会发展为肝癌,而蛋白组和代谢组数据也表明循环脂质代谢物以及一些基因的变化与该进程密切相关^[6]^。因此NASH作为严重肝脏疾病发生的必经阶段,了解其疾病发生发展及炎症恶性转化过程中相关生物分子调控的关系,对开展有效治疗严重肝脏疾病具有重要意义。目前营养诱发动物模型是展开NASH发病机制和治疗研究的最有效平台,如基于蛋氨酸和胆碱缺乏(methionine choline-deficient, MCD)饲料诱导的小鼠NASH模型。

LAMTOR1(全称为晚期胞内体/溶酶体接头蛋白,MAPK以及mTOR激活蛋白1)在调控细胞生长和能量平衡中起重要作用,并参与氨基酸介导的蛋白激酶复合物mTORC1的激活过程^[7]^。并在肝脏特异性敲除*LAMTOR1*的小鼠模型中发现,在能量匮乏时该蛋白参与重要蛋白激酶AMPK的激活^[8]^,以应对机体内的营养压力,表明该蛋白与肝脏的糖脂代谢关系密切^[9]^。因此,推断LAMTOR1在NASH疾病发展乃至后续进展为肝癌的过程中可能调控关键代谢通路,占据重要角色。

随着近些年功能代谢组学^[10]^的兴起,代谢组学技术在研究基因与疾病进展相关功能时发挥着越来越出色的作用^[11,12]^。将基因敲除和代谢组学技术联合交叉应用到生命过程中重要代谢通路的研究中,更利于阐释代谢疾病进展过程中的分子机制。本研究以MCD饮食诱导的小鼠NASH模型为平台,在肝脏特异性敲除*LAMTOR1*基因的组织样品中进行关键代谢通路鉴定,结合基因表达相关性分析,期望在NASH疾病进程及肝脏炎症恶性转化中对LAMTOR1蛋白可能发挥的重要作用给出阐释,并为NASH及NASH转化的肝癌发病机制和治疗研究提供理论依据。

## 1 实验部分

### 1.1 仪器与试剂

代谢物分析采用Acquity超高效液相色谱(UHPLC)(美国Waters)联合四极杆-飞行时间质谱(TripleTOF 5600 MS)(美国AB SCIEX)系统。蛋白质免疫印迹实验(western blot, WB)在Fusion Fx化学发光仪(法国VILBER)上实现。液相色谱用纯甲醇和乙腈购自Merck(德国),超纯水由Milli-Q系统制备(美国Millipore)。流动相添加剂甲酸和碳酸氢铵以及提取剂甲基叔丁基醚(methyl tert-butyl ether, MTBE)购自Sigma公司(美国),稳定同位素内标均为Sigma公司产品。稳定同位素内标有:亮氨酸-d3、苯丙氨酸-d5、色氨酸-d5、乙酰肉碱-d3、癸酰基肉碱-d3、棕榈酰肉碱-d3、硬脂酸-d3、软脂酸-d3、胆酸-d4和鹅去氧胆酸-d4。Tris-Cl、EDTA、5% (v/v) NP-40水溶液、脱氧胆酸和5% (v/v) Triton水溶液均购买自索莱宝(中国)。NaCl和甘油购买自科密欧(中国)。用于WB实验的放射免疫沉淀测定(radioimmunoprecipitation assay, RIPA)裂解液包含50 mmol/L Tris-Cl (pH=7.4)、150 mmol/L NaCl、1% (v/v) NP-40、0.25% (w/v)脱氧胆酸、10% (v/v)甘油、1 mmol/L EDTA (pH=8.0)和1% (v/v) Triton,使用时按照裂解液体积加入1% (w/v)蛋白酶和磷酸化抑制剂(美国InvivoGen)。兔抗LAMTOR1多抗为厦门大学林圣彩课题组制备(1-64aa)。兔抗p70S6激酶(p70S6K, cat #2708)、兔抗磷酸化p70S6激酶(phospho-p70S6K, cat #9234)、鼠抗S6核糖体蛋白(S6, cat #2317)、兔抗磷酸化S6核糖体蛋白(phospho-S6, cat #2215)和兔抗磷酸化4E-BP1(phospho-4E-BP1, cat #9955)等抗体购自CST公司(美国)。鼠抗GAPDH单抗(60004-1-Ig)和鼠抗Vinculin单抗(v4505)分别购自美国Proteintech和Sigma公司。

### 1.2 动物培养与组织苏木精-伊红染色

肝脏特异性敲除*LAMTOR1*的小鼠(*LAMTOR1*^LKO^, KO小鼠)由厦门大学林圣彩课题组采用Cre-LoxP重组酶系统获得^[10]^,并完成MCD饮食诱导的KO小鼠和野生型小鼠(WT小鼠)的NASH造模实验、肝脏组织取样及小鼠肝脏切片的苏木精-伊红染色(hematoxylin-eosin, HE)染色实验。

### 1.3 WB分析

在肝脏特异性*LAMTOR1*敲除KO小鼠和WT小鼠的肝脏组织中加入RIPA裂解液,经由氧化锆小珠在组织研磨器(中国新芝生物科技公司)上获得组织浆液,经过4 ℃、12000 r/min离心15 min后,取上清进行蛋白定量并调平蛋白浓度后,加入上样缓冲液变性。使用SDS-聚丙烯酰胺凝胶电泳进行蛋白分离。分离完全后将蛋白转至PVDF(polyvinylidene fluoride)膜上,并用一抗4 ℃孵育过夜,二抗室温孵育1 h。最后,通过化学发光试剂检测蛋白表达情况。

### 1.4 小鼠肝脏组织代谢物提取

代谢物的提取采用基于MTBE的液液萃取方法进行^[13]^并根据实验体系稍作改动^[14]^。称取约10 mg新鲜组织,置于加有氧化锆小珠的圆底离心管中,加入含有内标的甲醇提取剂后用球磨仪(mixer mill MM400,德国)进行组织匀浆,随后加入MTBE在室温下振荡。之后加入纯水涡旋振荡后静置分层。在10000 r/min、4 ℃条件下进行10 min高速离心使两相分离。按照5;3比例^[13]^分别取上层和下层清液,混合用于代谢组学分析。制备质量控制(quality control, QC)样本时,从每个提取样本中等量取上层和下层清液,分别置于两个离心管中,涡旋,同样5;3的比例分别取上层和下层清液制备QC样本。

### 1.5 LC-MS分析

LC-MS分析条件参考文献所述^[15]^。样品分析前用20% (v/v)甲醇水溶液进行复溶,正离子模式下,样品经过Acquity C8色谱柱(100 mm×2.1 mm, 1.7 μm)分离,柱温40 ℃,进样量5 μL。流动相A为0.1% (v/v)甲酸水溶液,流动相B为0.1% (v/v)甲酸乙腈。洗脱梯度:0~0.5 min, 5%B; 0.5~24 min,由5%B线性升至100%B; 24~28 min, 100%B;然后在0.1 min内回到初始比例5%B并平衡色谱柱1.9 min。流动相流速为0.35 mL/min。

负离子模式下,采用Acquity T3色谱柱(100 mm×2.1 mm, 1.8 μm),柱温50 ℃,进样量5 μL。流动相C为溶解6.5 mmol/L碳酸氢铵的水溶液,流动相D为含有6.5 mmol/L碳酸氢铵的95% (v/v)甲醇水溶液。洗脱梯度如下:0~1 min, 2%C; 1~22 min,由2%C线性升至100%D; 22~26 min, 100%D;然后在0.1 min内回到初始比例2%D并平衡色谱柱3.9 min,流动相流速度为0.35 mL/min。

质谱分析在全扫获得一级谱图的同时进行数据相关采集模式获得二级谱图。参数设置如下:离子源温度为500 ℃,离子源电压在正负离子分析模式下分别设为5500 V和-4500 V,去簇电压在正负离子模式下分别为100 V和-100 V,碰撞能在正负离子法分析模式下分别为30 eV和-10 eV。

### 1.6 数据处理和统计分析

将代谢组学平台采集的原始数据导入仪器自带的峰匹配软件Markerview (1.2.1版本,美国AB SCIEX公司)工作站中获取离子原始峰表。基于精确质量数、保留时间和MS/MS图谱^[16]^,以及在Peakview (1.2版本,美国AB SCIEX公司)工作站中提取二级碎片信息进行核对,进一步结合标样库、网络数据库等对代谢物进行定性。最后在Multiquant (2.1版本,美国AB SCIEX公司)中实现对定性代谢物分子的峰面积提取。代谢组学分析得到的原始峰面积,经由内标峰面积和组织重量校正后进行处理和统计分析。统计学显著性采用R语言中的Wilcoxon, Mann-Whitney test进行评价,将*p*<0.05的代谢物归为显著性的差异代谢物。组间存在差异的代谢物,数据归一化以后在Multi Experiment Viewer 4.8.1软件上热图可视化并进行层次聚类分析。并在开源的MetaboAnalyst网站(http://www.metaboanalyst.ca)进行差异代谢物的通路富集分析。使用软件SIMCA 14.0 (Umetrics,瑞典)进行主成分分析(principal component analysis, PCA),呈现样品间代谢特征的差异以及实验数据的稳定性。

## 2 结果与讨论

### 2.1 公共基因组数据库中分析*LAMTOR1*基因在NASH和肝癌中的表达情况

已有的报道^[7,9]^虽然显示出*LAMTOR1*基因在机体内的重要调控作用,但是其与NASH或者炎症恶性转化成的肝癌的疾病发生发展是否存在调控关系却未有明确的机制阐释。根据来源公共开放基因表达数据库Gene Expression Omnibus (GEO)中的一个数据集GDS4881 (https://www.ncbi.nlm.nih.gov/geo/query/acc.cgi?acc=GSE48452),通过基因表达数据的分析比较,我们发现相对于健康人样本(*n*=27), *LAMTOR1*在NASH病人样本(*n*=18)中存在高表达趋势(见图1a)。接下来基于TCGA (The Cancer Genome Atlas)数据库中的肝癌相关基因表达数据,我们在GEPIA (Gene Expression Profiling Interactive Analysis, http://gepia.cancer-pku.cn/)上进行了*LAMTOR1*的差异表达分析。通过对比TCGA肝癌数据库的正常样本(*n*=50)数据,我们发现*LAMTOR1*在肝癌的癌症样本(*n*=369)中是明显高表达的(见图1b)。根据*LAMTOR1*在NASH和肝癌中均出现高表达的异常现象,推测其在NASH的疾病进程乃至后续发展为严重的肝癌过程中,可能发挥重要的生物学调控功能。

**图1:**
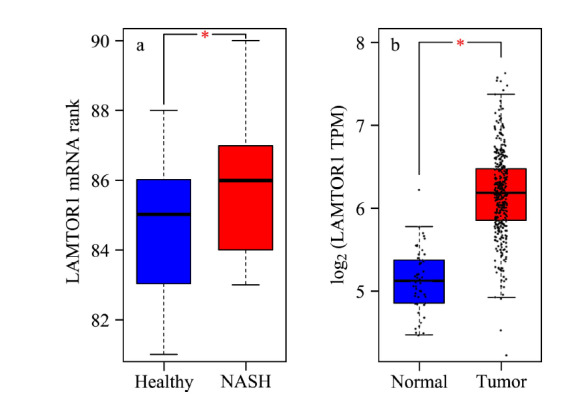
基于基因表达数据库分析*LAMTOR1*基因在 NASH和肝癌中的表达情况

### 2.2 小鼠NASH模型的成功构建与蛋白表达验证

为了模拟肝脏发生炎症性损伤的情况,用MCD饲料喂养诱导小鼠构建NASH模型,通过HE(hematoxylin-eosin)染色,观察到无论是肝脏特异性敲除*LAMTOR1*基因的小鼠(见图2a)还是野生型小鼠(见图2b)的肝脏组织中均出现较为明显的脂质沉积,并且镜下观察到肝细胞气球样变以及一些炎症细胞聚集的现象,这些特征的出现说明小鼠NASH模型的成功构建^[17]^。同时又提取了KO小鼠和WT小鼠肝脏组织中的蛋白,并进行了WB分析。如预期一样,在KO小鼠的肝脏组织中,由于*LAMTOR1*的敲除,LAMTOR1蛋白也不再表达(见图3a)。根据现有文献报道,LAMTOR1与其他复合物蛋白共同对mTOR通路及其下游蛋白的影响^[18]^也得到了验证,比如磷酸化p70S6k蛋白水平(见图3a)和磷酸化核糖体蛋白S6蛋白表达水平(见图3b)在*LAMTOR1*敲除小鼠肝脏中明显下调,而磷酸化4E-BP1^[19]^的蛋白水平发生明显上调(见图3b)。

**图2:**
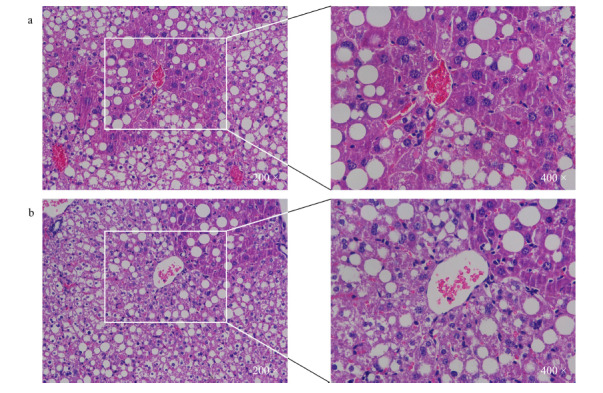
诱导NASH模型后*LAMTOR1*^LKO^小鼠和WT小鼠肝脏组织的苏木精-伊红染色结果

**图3:**
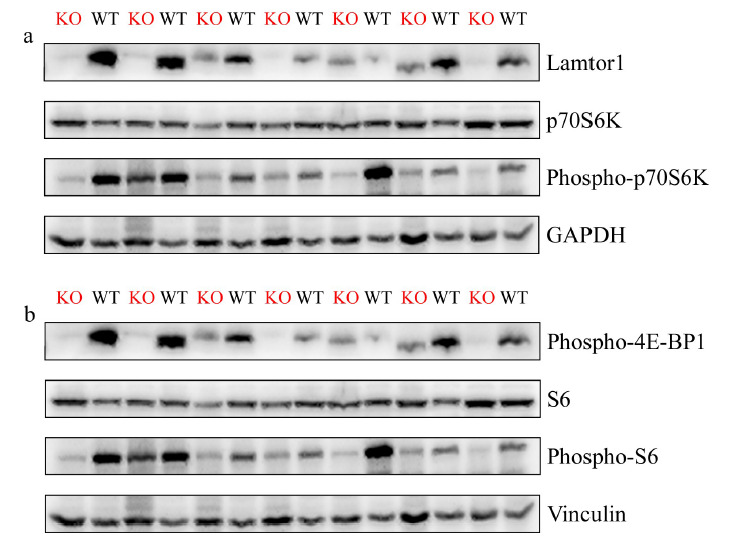
免疫印迹分析诱导NASH模型后*LAMTOR1*^LKO^小鼠和WT小鼠肝脏组织中LAMTOR1和受LAMTOR1调控的蛋白质水平

这些已知信号通路相关蛋白的变化说明在小鼠发生肝脏炎症性损伤时,*LAMTOR1*基因参与调控糖脂代谢,应对机体营养压力的功能仍然存在。解释清楚MCD诱导的小鼠NASH模型中,肝脏特异性敲除*LAMTOR1*具体通过调控哪些重要代谢通路来参与NASH进展中的肝脏功能调节,对理解NASH的疾病发生发展机制及预防治疗具有重要指导意义。

### 2.3 诱导NASH模型后*LAMTOR1*^LKO^小鼠和WT小鼠肝脏组织的代谢差异

本研究采用已建立的基于LC-MS的非靶向代谢组学方法对小鼠肝脏代谢物进行分析,该方法建立以后在许多疾病研究中得到了广泛应用^[15,20]^,其建立及考察过程参见文献^[21]^。在正离子模式和负离子模式下共定性到134个代谢物。用质控样本QC中代谢物的RSD分布来评价该方法在本研究中的重复性,其中正离子模式下检测的88个代谢物中,58个(65.9%)代谢物分子的RSD小于10%, 25个代谢物分子的RSD在10%~20%之间,5个代谢物分子的RSD在20%~30%之间(见图4a);负离子模式下定性到的46个代谢物分子的RSD均小于30%(见图4b),说明该代谢物分析方法在本研究中得到的数据可靠。对诱导NASH模型后*LAMTOR1*^LKO^小鼠肝脏组织、WT小鼠肝脏组织以及QC样本中检测到的代谢物进行了主成分分析。PCA模型(*R*^2^*X*=0.763, *Q*^2^=0.235)得分图(见图4c)中,QC样本紧密地聚集在一起,说明该液相色谱-质谱联用方法分析这些小鼠肝脏样品的结果稳定且重复性好。并且NASH模型下*LAMTOR1*^LKO^小鼠和WT小鼠肝脏组织之间有明显的分离趋势,说明在NASH模型中,肝脏特异性敲除*LAMTOR1*对小鼠肝脏的代谢过程有明显的扰动。从NASH小鼠肝脏代谢物的火山图(见图4d)可以看出,与WT小鼠相比,*LAMTOR1*^LKO^小鼠肝脏中有34个代谢物明显上调,11个代谢物发生明显下降。接下来对两组之间的差异代谢物进行热图可视化分析(见图5a),结果显示一些氨基酸、脂肪酸等代谢物在肝脏特异性敲除*LAMTOR1*小鼠肝脏中差异显著上调,反而*N*-乙酰谷氨酸、精胺等代谢物显著下调。对差异代谢物做通路富集分析(见图5b),结果显示NASH模型下*LAMTOR1*^LKO^小鼠肝脏中嘧啶代谢、核黄素代谢、精氨酸生物合成、谷胱甘肽代谢、氨基酰-tRNA生物合成、氮代谢、谷氨酰胺和谷氨酸代谢和beta-丙氨酸代谢等通路发生不同程度的扰动。

**图4:**
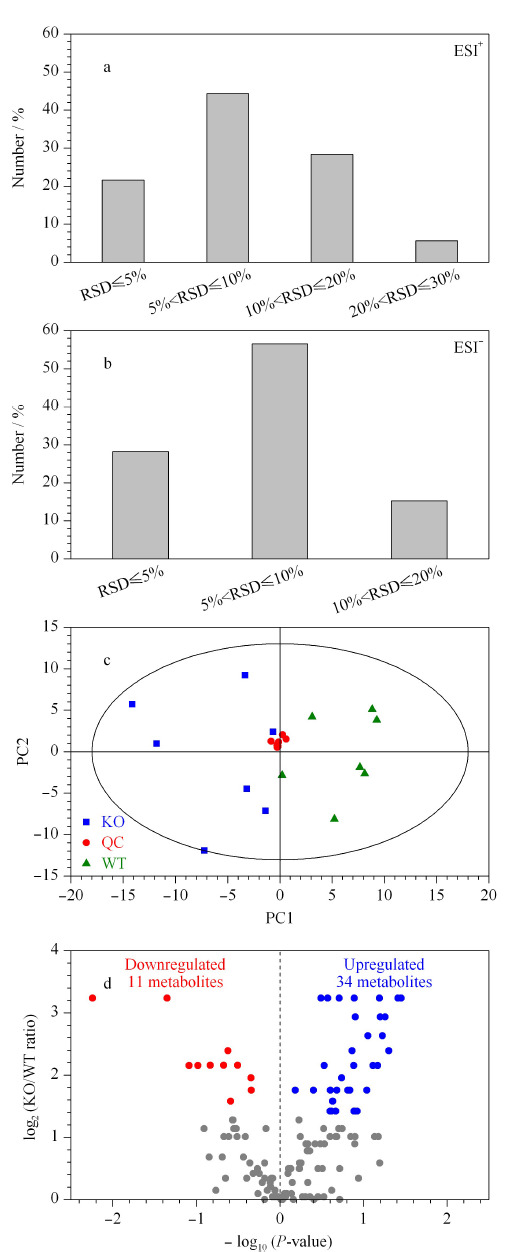
(a)正、(b)负模式下QC样本中代谢物的RSD分布及诱导NASH模型后*LAMTOR1*^LKO^小鼠和WT小鼠肝脏组织代谢物的(c)主成分分析图和(d)火山图

**图5:**
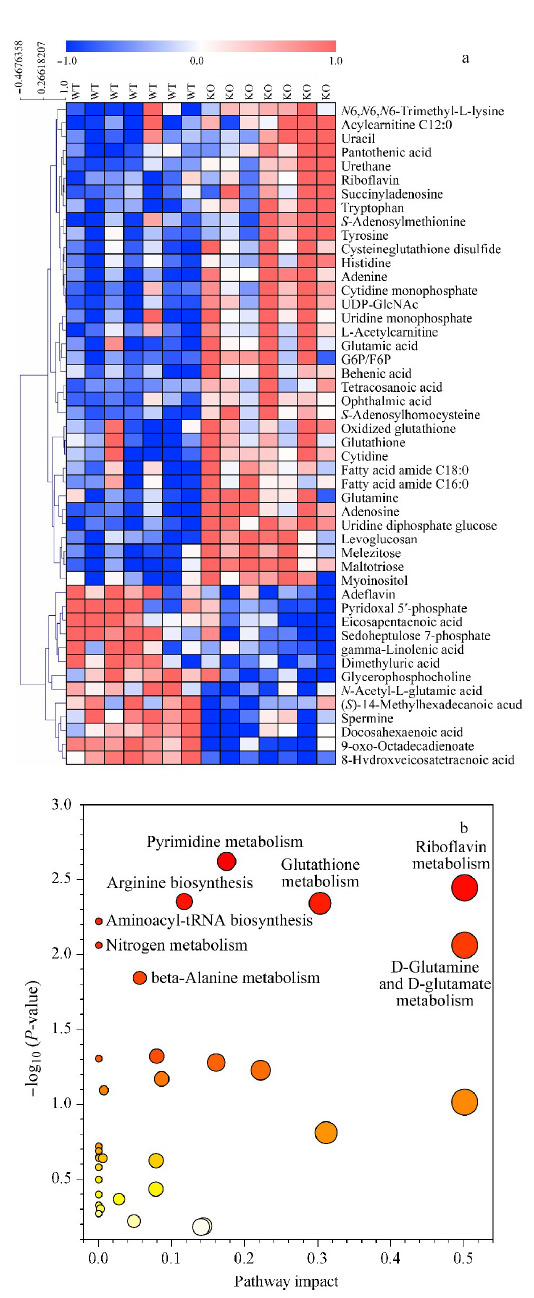
诱导NASH模型后*LAMTOR1*^LKO^小鼠和WT小鼠肝脏组织中差异代谢物(*n*=7)的(a)热图和(b)通路分析

### 2.4 *LAMTOR1*基因在肝脏炎症恶性转化中调控的代谢通路鉴定与分子机理

代谢组学结果分析发现,当小鼠肝脏发生炎症性损伤时己糖胺生物合成通路中的中间代谢产物谷氨酰胺、谷氨酸、UDP-GlcNAc等在*LAMTOR1*^LKO^小鼠肝脏中明显上调(见图6a)。已知机体内己糖胺的生物合成通路是氨基糖和核苷酸糖代谢的重要分支^[22]^,该通路合成的终产物UDP-GlcNAc在后续的蛋白糖基化修饰中发挥重要作用,在癌症^[23]^等疾病中参与线粒体、细胞质和细胞核内的相关蛋白的表达和功能调节。在*LAMTOR1*^LKO^小鼠肝脏中UDP-GlcNAc发生累积,推测可能是己糖胺的生物合成通路被激活,或者可能是蛋白糖基化过程对该化合物的利用受到阻碍。由于公共开放数据库中NASH相关表达数据有限,接下来我们基于TCGA数据库中肝癌相关样本和GTEx (Genotype-Tissue Expression)中肝脏组织的基因表达数据,对LAMTOR1与糖基转移酶之一MGAT1的基因表达情况进行Pearson相关系数分析。得到的相关曲线结果见(见图6b),在肝癌中*LAMTOR1*基因与*MGAT1*基因可能存在正调控的关系(*R*=0.47),启示我们在小鼠NASH模型中,肝脏中*LAMTOR1*的敲除可能通过调控糖基转移酶MGAT1的表达减少或者酶活降低,造成上游底物UDP-GlcNAc累积的情况出现。那么在NASH的疾病进展中,LAMTOR1与己糖胺合成产物的利用以及后续一些糖基转移酶的调控关系则需要进一步的研究。

**图6:**
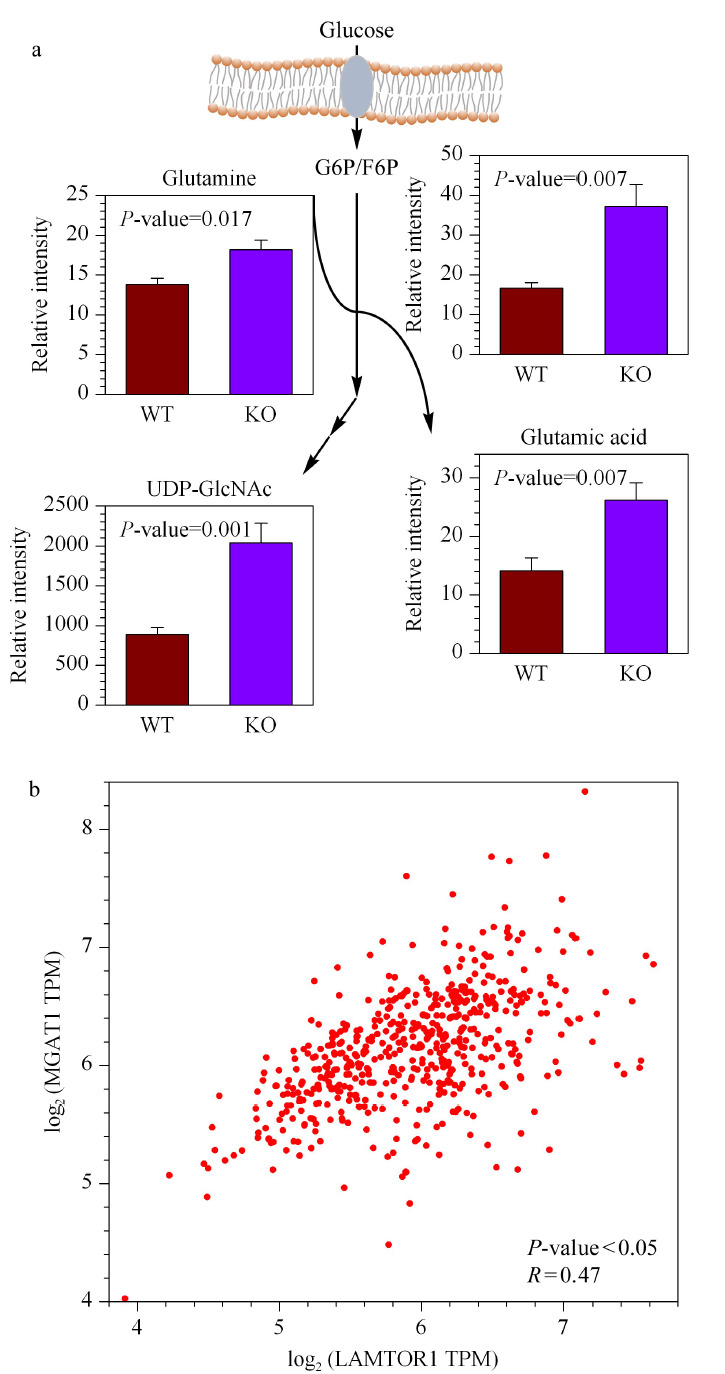
表征*LAMTOR1*在小鼠NASH模型(*n*=7)中对己糖胺生物合成通路的影响并分析肝癌里*LAMTOR1*与*MGAT1*的基因表达相关性

同时,在KO小鼠的肝脏中也观测到了甲基化的重要甲基供体——代谢物*S*-腺苷蛋氨酸(*S*-adenosylmethionine, SAM)的上调。SAM可在甲基转移酶的作用下将甲基转移给DNA和蛋白等生物分子,同时生成*S*-腺苷高丝氨酸(*S*-adenosylhomocysteine, SAH)。除了SAH、SAM以外,在赖氨酸残基上反应生成的产物三甲基赖氨酸也在KO小鼠的肝脏中明显上调(见图7)。同样,对肝癌里*LAMTOR1*与蛋氨酸腺苷转移酶*MAT1A*的基因表达情况,进行Pearson相关性分析。分析发现在肝癌中*LAMTOR1*基因与*MAT1A*基因可能存在负调控的关系(*R*=-0.47)(见图7),说明在肝癌的进展中LAMTOR1的低表达可能会促进蛋氨酸腺苷转移酶MAT1A的高表达或者酶活上调,从而促进甲基化供体SAM的产生。上述结果启示在小鼠NASH模型中,*LAMTOR1*也可能通过对SAM产生的相关代谢酶的调控来影响小鼠NASH的进程及炎症恶性转化。

**图7:**
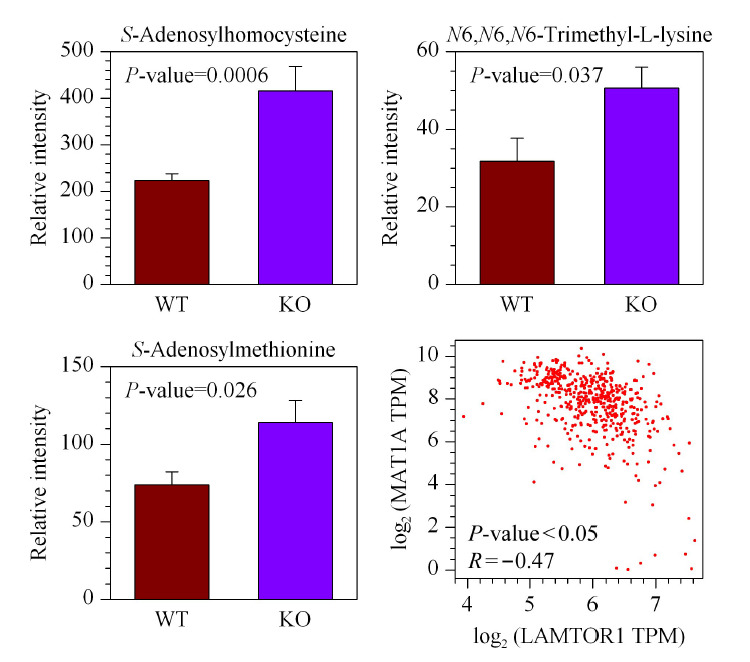
*LAMTOR1*在小鼠NASH模型中引起蛋白甲基化相关代谢物的变化和肝癌里*LAMTOR1*与*MAT1A*的基因表达相关性分析

另外,重要功能代谢物琥珀酰基-腺苷(succinyladenosine, SAdo)在*LAMTOR1*敲除的小鼠肝脏中也发生明显的上调(见图8a)。据文献报道,腺苷基琥珀酸裂解酶ADSL缺乏的情况下机体内会出现SAdo的累积^[24]^。在肝癌数据库中进行Pearson相关性分析发现,*LAMTOR1*与*ADSL*基因之间存在正向调控的关系(*R*=0.59)(见图8b)。因此肝脏中*LAMTOR1*敲除可能会阻碍ADSL对于代谢物SAdo的降解,上调的SAdo水平将对小鼠肝脏的代谢调控产生影响从而参与到NASH的疾病进程及后续的炎症恶性转化中。此外,9-氧代十八碳二烯酸、二十碳五烯酸(eicosapentaenoic acid, EPA)和二十二碳六烯酸(docosahexenoic acid, DHA)等不饱和脂肪酸和甘磷酸胆碱(glycerophosphorylcholine, GPC)在肝脏特异敲除*LAMTOR1*的NASH模型小鼠肝脏组织中也显著下降(见图9)。

**图8:**
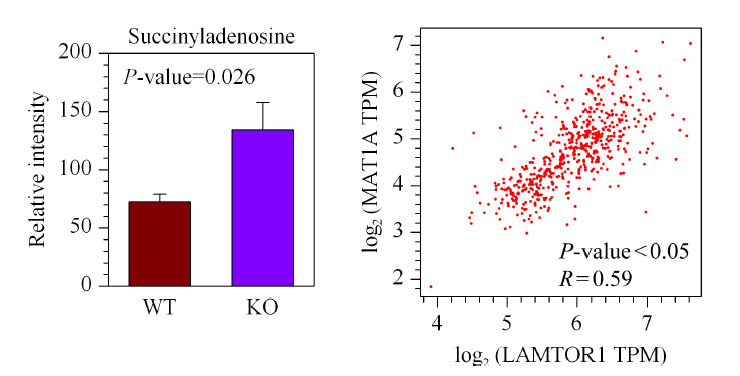
诱导NASH模型后*LAMTOR1*^LKO^小鼠和WT小鼠肝脏组织中的琥珀酰腺苷水平变化(*n*=7)和肝癌里*LAMTOR1*与*ADSL*的基因表达相关性分析

**图9:**
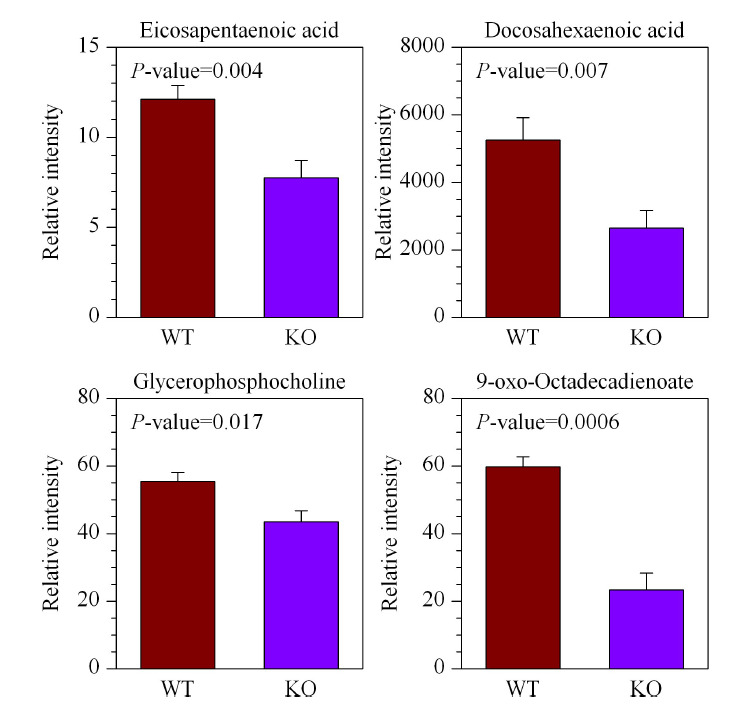
4个炎症相关代谢物在诱导NASH模型后*LAMTOR1*^LKO^小鼠和WT小鼠肝脏组织中的变化(*n*=7)

而已有的文献报道NAFLD中不饱和脂肪酸水平的降低与肝脏疾病的进展程度相关^[25]^,并检测了肝脏中相关基因的表达情况^[26]^,其中发现相对于单纯脂肪变性样本,NASH样本中EPA和DHA占据肝脏总脂质的比例下降,说明这两个不饱和脂肪酸水平的降低对于NASH的进展可能有促进作用。本研究建立的小鼠NASH模型中,肝脏特异敲除*LAMTOR1*的小鼠肝脏中EPA和DHA显著下降,说明*LAMTOR1*基因的敲除在小鼠NASH的进程中可能发挥一个消极地促进作用。同时,本研究中发现肝脏特异敲除*LAMTOR1*的NASH小鼠肝脏中GPC水平降低。此外,基于核磁共振磷谱技术的NAFLD病人肝脏代谢结果^[27]^,具有NASH相关症状的NAFLD病人相比无NASH特征的病人,肝脏中GPC水平降低。因此,可以推断*LAMTOR1*在NASH发生发展中起重要作用。

## 3 结论

本研究用MCD饮食诱导小鼠发生肝脏炎症性损伤,以此NASH疾病模型为平台,借助基于液相色谱-质谱联用的代谢组学手段,鉴定了肝脏特异性敲除*LAMTOR1*在小鼠肝脏中调控的重要代谢通路,并结合现有公共开放组数据库进行预测分析,研究了*LAMTOR1*在NASH疾病进展乃至发展为更严重的肝癌过程中可能参与的分子调控机理。其中重要代谢物水平的变化、*LAMTOR1*与其他相关基因在肝癌中相关性预测结果都显示LAMTOR1在NASH疾病及后续的炎症恶性转化中可能发挥着重要作用。但是,肝脏炎症恶性转化中LAMTOR1与鉴定到的关键代谢通路的直接关系还需要进一步的研究。
